# Prevalence of *Moraxella Catarrhalis* as a Nasal Flora among Healthy Kindergarten Children in Bhaktapur, Nepal

**DOI:** 10.1155/2022/3989781

**Published:** 2022-03-26

**Authors:** Neetu Amatya, Govinda Paudel, Bhuvan Saud, Sunita Wagle, Vikram Shrestha, Bibhav Adhikari

**Affiliations:** ^1^Department of Medical Laboratory Technology, Janamaitri Foundation Institute of Health Sciences (JFIHS), G.P.O. Box 8322, Kathmandu, Nepal; ^2^Dhading Hospital, Dhading, Government of Nepal, Kathmandu, Nepal; ^3^Little Angles' College of Management, G.P.O. Box 8322, Hattiban, Lalitpur, Nepal

## Abstract

**Introduction:**

*Moraxella catarrhalis* causes mild to severe disease in all age groups, mainly in children. This study investigates the prevalence of *M. catarrhalis*, its cocolonization with other common nasal flora, and associated risk factors in kindergarten children in Bhaktapur.

**Method:**

A cross-sectional study was conducted among 136 healthy school-going children from four kindergartens of Bhaktapur Municipality. Nasal swabs were examined for identification and isolation of *M. catarrhalis* and its antibiotic susceptibility pattern. Additionally, further analysis was performed for cocolonization and associated risk factors.

**Results:**

Out of 136 students, *M. catarrhalis* was detected in 80 (58.8%) children. Using bivariate and multivariate analysis, the associated risk factors with significantly high carriage rates were age group of 3–4 years, classroom occupancy with 15–30 children, and antibiotic consumption within 6 months, with a *p* value of ≤0.05 in each of the cases. Multiple logistic regression analysis of bacterial coexistence depicted *M. catarrhalis* to be positively associated with *Streptococcus pneumoniae* and *Haemophilus influenzae* and negatively associated with *Staphylococcus aureus*. Furthermore, the highest double colonization occurred among *M. catarrhalis* and *S. aureus* and the highest triple colonization occurred among *M. catarrhalis*, *S. aureus*, and *S. pneumoniae*. The antibiogram pattern showed the target organisms to be highly resistant to amoxycillin/clavulanate (18.8%) and most sensitive to chloramphenicol (100%).

**Conclusion:**

This study shows a high prevalence of *M. catarrhalis* in healthy kindergarten children and is positively associated with other nasal isolates like *S. pneumoniae* and *H. influenzae*.

## 1. Introduction

*M. catarrhalis* is a Gram-negative diplococcus with flat adjacent sides [1]. This genus which currently comprises 16 different species was previously considered a harmless bacterium of the upper respiratory tract [2,3]. However, recent research studies suggest that it has been emerging as an important pathogen and has even been considered a well-established pathogen of the upper and lower respiratory tract in many parts of the world [4].

*Moraxella catarrhalis* is an important pathogen of upper respiratory tract infections and is most commonly associated with sinusitis and acute otitis media (AOM) [5]. It is the third leading cause of AOM (10.0 to 20.0%) following *Streptococcus pneumoniae* and nontypeable *Haemophilus influenzae* and the second important otopathogen detected in otitis media with effusion (9.0% to 24.0%) [6–8] and may cause comparatively decreased erythema and distortion of the tympanic membrane than *Streptococcus pneumoniae* infection [7]. Studies also reveal that *M. catarrhalis* is an important bronchopulmonary pathogen [9]. It is known to cause an estimated case of about 2–4 million exacerbations of chronic obstructive pulmonary disease per year in adults in the United States alone [10]. Moreover, it has been found to be the causative agent in infections, such as empyema, pneumonia, and even several cases of endocarditis, both in children and adults [5, 11].

Colonization of *M. catarrhalis* in healthy individuals is facilitated by various factors such as age, family size, socioeconomic status, vaccination status, and seasonal variation. It has a significant association with age as the colonization seems to be higher in children than in adults [12–15]. Evidence regarding *M. catarrhalis* virulence properties is inconsistent in various studies [16]. However, recent research studies have mentioned that the associated virulence factors are whole bacteria or their outer membrane vesicles that aid colonization, infection, and antibody formation [17]. Unlike other pathogens, it is generally susceptible to many antibiotics; however, newer drug resistance is emerging [18]. The emergence and extensive spread of antimicrobial resistance in the natural microbial community are poorly understood [19]. Recent findings have suggested that penicillin resistance is mediated by the formation of beta-lactamases encoded by the genes bro-1 and bro-2 [20].

The rate of *M. catarrhalis* detection has marked up to 30%, consequently making the bacterium the second most common cause of AOM and otitis media with effusion. Since the introduction of the pneumococcal conjugate vaccine (PCV) with/without protein D of nontypeable *H. influenzae*, *M. catarrhalis* has now been regarded as a high-priority pathogen responsible for otitis media, as several studies have shown a shift of otopathogens in AOM. Because there are no vaccines commercially available, research studies strive to seek novel targets for vaccine development against *M. catarrhalis* [7]. Globally, *M. catarrhalis* has been established as an important emerging pathogen, especially in children [16]. In a study done in Nepal, *M. catarrhalis* was detected in 6.90% of sputa cultures [17].

In Nepal, limited studies have been conducted to determine the prevalence, antibiotic resistance pattern, and associated risk factors of *M. catarrhalis*. In an economically deprived country like Nepal, children are brought up in a relatively poorer quality environment and hence are especially vulnerable to respiratory tract infections. Our study, therefore, targets to investigate the prevalence, risk factors, and antibiogram of nasal carriage of *M. catarrhalis* in healthy kindergarten children and understand its co-existence with other common nasal bacterial flora in healthy kindergarten children vaccinated with PCV (pneumococcal conjugate vaccine) in Bhaktapur Municipality, Nepal.

## 2. Materials and Methods

### 2.1. Study Design and Site

In this cross-sectional study, kindergarten children of 2 schools and 2 daycare centers of Bhaktapur Municipality were selected through convenient sampling from May 2018 to October 2018. The study was conducted in the Department of Medical Laboratory Technology, Janamaitri Foundation Institution of Health Sciences, Hattiban, Lalitpur. Manuscript writing was carried out as per the standard IMRAD (introduction, methods, results, and discussion) format [18].

### 2.2. Study Population and Criteria

A total of 136 children were enrolled in this study. A set of questionnaires was sent to the parents/guardians of children aged 2–5 years, one week prior to sample collection. The questionnaires included various parameters such as demographic information, medical information, history of vaccination, respiratory illness in the past 6 months, antibiotic consumption within 7 days of enrolment, immunodeficient condition, and parents' education status. Furthermore, other study variables included were age, gender, mother's education and father's education, location of kindergarten, occupancy of classes, and grade and category of school. Children aged 2–5 years old with parental consent were short-listed as an inclusion population in this study. Nevertheless, exclusion criteria encompass those with acute respiratory symptoms (<72 hrs. of onset), any sort of respiratory tract infection or nasal abscesses, antibiotic consumption within 7 days of enrolment, immunodeficient conditions, any unfit physical condition for swab collection, or those children without consent.

### 2.3. Sample Collection and Processing

A sterile cotton swab was moistened by dipping it into sterile distilled water and pressing it on the inner wall of the bottle to drain the excess water. A nasal swab was collected by introducing the moist cotton swab into the nostril, parallel to the palate, and letting it place inside for a few seconds. The swab was then withdrawn slowly with a rotating motion. The same procedure was carried out in the other nostril with the same cotton swab. Soon after the collection of the nasal swabs; it was packed in a sterile test tube, labeled properly, and then transported to the laboratory and processed within half an hour of sample collection. Inoculation was carried out in blood agar, mannitol salt agar, and chocolate agar with bacitracin (10 units). Blood agar and chocolate agar were placed in a candle jar while mannitol salt agar was placed in normal atmospheric conditions. All the plates were incubated at 37°C for 48 hours.

### 2.4. Microbial Identification and Sensitivity Test

Identification of *M. catarrhalis* and other bacteria was carried out by colony morphology, Gram staining, and other relevant biochemical tests. Antimicrobial susceptibility testing of the isolates was performed by the Kirby–Bauer disc diffusion method [19]. A detailed list of biochemical tests performed is shown in Table 1.

### 2.5. Statistical Analysis

All participants were encoded. SPSS (Statistical Package for the Social Sciences) version 21 was employed in analyzing the data. The comparison of categorical variables was evaluated by the chi-square test. A multiple logistic regression model was used to test the association between demographic characteristics and bacterial coexistence. Significant level was set at significant level *p* < 0.05.

## 3. Result

### 3.1. Sociodemographic Study and Clinical Information

This study comprises a total of 136 participants (male = 81 (59.6%) and female = 55 (40.4%)). The mean age of the participants was 3.2 years. As shown in detail in Table 2, most of the parents were educated. The classroom with the occupancy of 15–30 children (51, 37.5%) has the highest frequency. Likewise, the majority of the children were from ward number 7 (*n* = 51, 37.5%). Maximum children (*n* = 81, 59.6%) did not consume any antibiotics within 6 months' interval. The details of the sociodemographic and clinical information are shown in Table 2.

### 3.2. Bivariate and Multivariate Analysis of Risk Factors for *M. catarrhalis*

Out of the total 136 participants, nasal carriage of *M. catarrhalis* was detected in 80 (58.8%) children.  Table 3 infers the bivariate and multivariate analysis of risk factors for carriage. It is found that the prevalence of the target isolates was higher in females (61.8%) than in males (56.8%). The difference, however, was statistically insignificant. Similarly, going through the age groups, the maximum carriage rate was seen in the age group of 4–5 years (76.9%), the difference being statistically significant when compared with the reference age group, p value 0.04 (O.R. = 0.04, C.I. = 0.15–0.94).

In the case of the category of kindergarten school chosen, children from government schools had a higher carriage rate of 62.1% compared to those from private schools (53.1%), the difference, however, being statistically insignificant, *p* value 0.3. Likewise, grade-wise analysis of children showed no statistical difference in the carriage rate of the target organism. The education level of parents was not found to have a significant impact on the carriage rate of the target bacterium. Similarly, classroom occupancy of 15–30 showed the highest prevalence (70.6%) of target bacteria with a statistically significant difference (p value 0.05) (O.R. = 2.4 and C.I. = 0.48–2.67). Study of prevalence on geopolitical location showed no statistically different carriage of the target isolate on the respective wards. Children who did not consume any antibiotics within 6 months had a significantly higher carriage rate (66.7%) with a p value of 0.03 (O.R. = 2.2 and C.I. = 1.1–4.5).

### 3.3. Multiple Logistic Regression Analysis of Bacterial Coexistence

Multiple logistic regression analysis showed *M. catarrhalis* was positively associated with other nasal isolates, namely, *S. pneumoniae* and *H. influenzae*. In contrary, it was negatively associated with *S. aureus*, the details of which are shown in Table 4.

**Figure 1:**
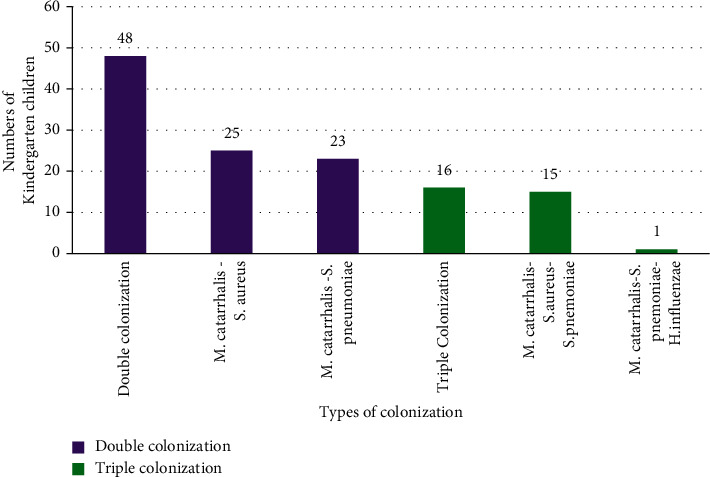
Colonization properties of *M. catarrhalis* with other carriers.

Figure 1 shows the double and triple colonization of *M. catarrhalis* along with other nasal isolates, where the highest double carriage included *S. aureus* and *M. catarrhalis* with a prevalence of 18.4%, and the highest triple colonization included *M. catarrhalis*, *S. aureus*, and *S. pneumoniae*.

### 3.4. Antimicrobial Activity of *M. catarrhalis*

**Figure 2:**
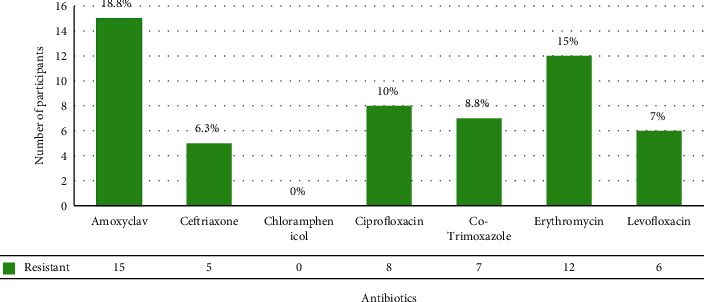
Antimicrobial resistance pattern of *M. catarrhalis.*

Figure 2 depicts *M. catarrhalis* to be the most resistant to Amoxyclav and most sensitive to chloramphenicol.

## 4. Discussion

*M. catarrhalis* causes severe respiratory tract infections, specifically in children, where the underlying respiratory symptoms are null in contrast to those of adults. It commonly causes sinusitis and acute otitis media along with other complications [5, 20].

The global carriage rate of *M. catarrhalis* is 16%–67% [21]. Our study showed a high prevalence of 62.5% among healthy school-going children. Similar findings were observed in the studies that were carried out in China (76.6%) [21], Belgium (67.0%) [22], Hungary (63.5%) [23], and South Sweden (52.1%) [24]. In contrast, the carriage rate in Korea was 35.0% [25]and in the Netherlands it was 25.8% [26]. This variation may be due to the carriage rate being affected by geographical and climatic factors [27]. A nuance decrement was found in the study carried out in another city of Nepal called Pokhara with a prevalence rate of 45.5% which may be due to environmental factors and variation in classroom occupancy when compared to our study [15].

Females in this study have a higher carriage of this bacterium when compared with males. The difference, however, is statistically insignificant. This finding is comparable with the findings of the studies conducted in the Netherland [28] and China [21].

This study depicts that the highest carriage rate was seen in the age group of 4–5 years (76.9%), while all age groups had a high carriage rate. Our findings are comparable to those of a study conducted in Belgium where young age (under 4 years) was a risk factor for *M. catarrhalis* carriage [22]. The prevalence of pathogens is generally higher in young age as their immunity is still immature [15]. Studies have shown that approximately 80.0% of children were diagnosed with otitis media by the age of 3 yrs [10] which is the most common bacterial infection seen in children and a dominant reason to prescribe antibiotics for the same. Meanwhile, a study in Pokhara showed the target organism was 38.8% in the age group 5–9 years which is less than the finding of our study [15].

In the case of category of schools, the public school has a dominant carriage rate of 62.1% compared to that of private schools; however, the difference is non-significant. Similar findings can be seen in a study of Pokhara, Nepal, that revealed government schools' children had the highest multiple bacterial carriage rate than those of private schools. The more specific reason behind this could be the overcrowding in the school [15] and the maximum occupancy of students in a single classroom. The bacterial carriage seems to fluctuate depending on the hygiene status and close peer-contact [21].

In our study, children from L.K.G. had the highest prevalence rate of 66.7%, followed by playgroup children (61.8%), and then 52.0% carriage in both nursery and UKG. In contrary, a study in Hungary showed a lucid decrease in carriage with age, i.e., 60.1% in nurseries, 37.6% in daycare centers, and 15.5% among school-children [23]. Many research studies have revealed that *M. catarrhalis* carriage decreases as age progresses and vice-versa [20, 21, 25, 28, 29]. However, there's a fluctuation in this trend in our study which is still unclear.

Scrutinizing into the colonization of *M. catarrhalis* with parents' education, the highest prevalence was seen in fathers pursuing bachelor's degree and above, whereas children with uneducated mothers had the greatest carriage of target bacteria. However, no significant effect of parents' education on the colonization rate has been found out. This result is comparable to that of Hungary [23] which also shows a similar non-significant association. Similarly, in Netherlands, colonization with *M. catarrhalis* was not associated with the educational level of the mother [28]. Albeit, the maximum carriage seen in children of uneducated mothers may be justified by the fact that in the case of Nepal, where mothers are mainly responsible to maintain the health, hygiene, sanitation, and care of their children, it is suggestive that the poor hygiene conditions of uneducated mothers could explain the higher carriage of *M. catarrhalis* in their children [21]. This logic is supported by a study in Belgium where children from lower socioeconomic backgrounds were more likely to carry nasal carriage [22].

On the basis of the occupancy of children in a classroom, the classroom with an occupancy of 15–30 seemed to have the highest prevalence of 70.6% and was strongly associated with a significant risk of target bacterial carriage (*p* = 0.05, OR = 2.4, and C.I. = 0.99–5.84). Here, overcrowding is one of the associated factors for the transmission among healthy children. The transmission and carriage rates are directly associated with the population where people live in close contact with one another, especially school children and those at home with siblings [15, 20]. Carriers can easily transmit the bacteria among siblings and among their peers if they are immune-compromised or have immature immunity causing community-acquired pneumonia, otitis media, acute sinusitis, and even an increased mortality rate in the worst situations.

The prevalence of *M. catarrhalis* carriage in various geopolitical locations (wards) studied within Bhaktapur had no statistically significant difference. This might be attributed to the fact that Bhaktapur, being the smallest district in Nepal, has less fluctuation in climatic and environmental conditions.

There was a significant difference in the bacterial carriage between children who had consumed the antibiotic within 6 months and those who had not (*P*=0.03, O.R. = 2.2, C.I. = 1.1–4.5) with the higher carriage seen in children without consumption of antibiotics (66.7%). Regardless of this result, other studies showed a non-significant association of bacterial carriage in children and previous antibiotic exposure [28]. Here, the factors that aid in the colonization of the pathogen are a prior history of respiratory infections and antibacterial exposure [21, 23].

In this study, a positive association of *M. catarrhalis* with *S. pneumoniae* and *H. influenzae* was seen, while a negative association was seen with *S. aureus*. This result exactly coincides with the studies in Indonesia [25] and Korea [30]. In contrary, *M. catarrhalis* was seen to be negatively associated with *H. influenzae* in other two studies [21, 31]. The reason for coexistence may occur when they are often found together rather than expected by chance [31]. This association may also be facilitated by the fact of interspecies quorum sensing, passive antimicrobial resistance, and host susceptibility [30]. Nevertheless, this interaction in terms of carriage and density may have consequence to clinical implications in a person too [32]. Furthermore, predicting the cause of the negative association of these bacterial species, it may occur when they compete in the same environment [31]. Other studies suggest that *S. aureus* is not only negatively associated with *M. catarrhalis* but also with *S. pneumoniae* and *H. influenzae* as well [23]. The mechanism of this repulsive nature is still dubitable, although the proposed mechanism may be by direct killing of other bacterial species via inhibitory effectors, namely, neuraminidase, peroxidase, and bacteriocin [25]. Additionally, the action of antimicrobials and vaccines for a specific bacterium may also alter the polymicrobial interaction among the nasopharyngeal species, having consequence for unanticipated results [25].

Nasopharyngeal cocolonization may promote the virulence property of the colonizing bacteria [20]. Here, in our study, double and triple cocolonization among *M. catarrhalis*, *S. pneumoniae*, *S. aureus*, and *H. influenzae* have been seen. A single triple cocolonization of *M. catarrhalis*, *S. pneumoniae*, and *H. influenzae* was reported. *M. catarrhalis*, *S. pneumoniae*, and *H. influenzae* are the three most common bacterial pathogens responsible for acute otitis media and for polymicrobial infections [21, 30]. Similarly, other findings have elucidated that *M. catarrhalis* and *H. influenzae* cocolonization enhances biofilm formation via quorum sensing and host clearance mechanisms [26]. There are several other factors that alter the carriage and cocolonization of respiratory pathogens such as age, geographical area, sampling site and collection technique, immunization program, socioeconomic condition, host-immunity, and environmental factors. [23, 31]. Therefore, a better understanding of the cocolonization properties and bacterial interaction is prominent to control the severity of upper respiratory tract infections [23].

In Nepal, PCV13 was introduced in regular basis from 18^th^ July, 2015 [33], whereas Hib vaccine was introduced in 2009 [34]. Thus, the commencement of these vaccination programmes against *S. pneumoniae* and *H. influenzae*, respectively, could have simultaneously reduced the colonization of the preceding two bacteria resulting *M. catarrhalis* to invade the gap previously colonized by other carriage and become the most frequent bacterial pathogen associated with otitis media in the future [10, 20, 35]. Thus, the prevalence of *M. catarrhalis* could be seen high in this study.

Further going, most of the isolates in this study were susceptible to antibiotics with 100% susceptibility to chloramphenicol, while the highest resistant to Amoxyclav (18.8%). This resistance to Amoxyclav in this study is in total contrast to other studies where Amoxyclav seems to have the highest activity with only 4.0% resistance in Taiwan [36], the United States [37], and 100% susceptibility in Hungary [17]. From this study, a slight increase in the resistance trend of Amoxyclav can be seen compared to other studies, potentially causing a threat to antibiotic resistance. Similarly, the worldwide prevalence of the bro gene in *M. catarrhalis* is seen in 95.0% of isolates in children. This bro-beta-lactamase gene also seems to indirectly benefit other colonizing bacteria, enhancing polymicrobial infection and facilitating treatment failure [38]. Furthermore, the continuous use of antimicrobial therapy for acute otitis media has resulted in rapid antibiotic resistance to other bacterial pathogens causing otitis media, limiting the clinical efficacy of antibiotics around the world [4]. The situation can get even worse in the future as, till date, neither a licensed vaccine against *M. catarrhalis* has been introduced nor has the putative vaccine been brought into clinical trial. Therefore, considering all these increasing antimicrobial resistance, morbidity, and mortality, a rapid response is of the utmost necessity, and the introduction of vaccines could be an alternative approach to prevent infection and minimize the excessive and unwise use of antibiotics.

## 5. Conclusion

Incidence of relatively rare pathogens like *M. catarrhalis* is common in children of Nepal where the mainstream medical protocol still being ignored. This study can be taken as a mere example of how children of under-developing countries like Nepal are at a high risk of respiratory tract-related infections.

## Figures and Tables

**Table 1:** Biochemical properties of isolated bacteria.

Biochemical Tests	Bacteria			
	*M. catarrhalis*	*S. aureus*	*S. pneumoniae*	*H. influenzae*
Catalase	**+**	**+**	-	**+**
Oxidase	**+**	-	-	ND
Lactose fermentation	-	+	ND	-
Nitrate reduction	**+**	+	ND	+
DNase	**+**	+	ND	ND
Mannitol fermentation	ND	+	ND	ND
Coagulase	ND	+	ND	ND
DNase	+	+	ND	ND
Nitrate reduction	+	+	ND	+
*α*- hemolysis	ND	ND	+	ND
Optochin (5µg) sensitivity	ND	ND	+	ND
Bile solubility	ND	ND	+	ND
Inulin fementation	ND	ND	+	ND
X (haemin) + V (NAD) factor requirement	ND	ND	ND	+
Satellitism test	ND	ND	ND	+

Note: + = positive; - = negative; ND = not done.

**Table 2:** Sociodemographic and clinical details of participants.

Characteristics of Children (n=136)		Numbers (%)
Gender	Male	81 (59.6)
	Female	55 (40.4)

Age	<3 yrs.	60 (44.1)
	3–4 yrs.	29 (21.3)
	4–5 yrs.	13 (9.6)
	5 yrs.	34 (25.0)

Category of school	Public school	87 (64.0)
	Private school	49 (36.0)

Grade	Playgroup	68 (50.0)
	Nursery	25 (18.4)
	L.K.G.	18 (13.2)
	U.K.G.	25 (18.38)

Father's education level	Primary	21 (15.4)
	Secondary	26 (19.1)
	Higher	39 (28.7)
	Bachelor and above	43 (31.6)
	Uneducated	7 (5.1)

Mother's education level	Primary	20 (14.7)
	Secondary	27 (19.9)
	Higher	41 (30.1)
	Bachelor's degree and above	35 (25.7)
	Uneducated	13 (9.6)

Classroom occupancy	<15	36 (26.5)
	15–30	51 (37.5)
	>30	49 (36.0)

Ward no.	10	48 (35.3)
	8	37 (27.2)
	7	51 (37.5)

Antibiotic consumption within 6 months	Yes	55 (40.4)
	No	81 (59.6)

U.K.G.: Upper kindergarten; L.K.G.: lower kindergarten.

**Table 3:** Bivariate and multivariate analysis of risk factors for carriage of *M. catarrhalis* among participants.

Parameters	Isolated		Not isolated		P value	OR (CI 95%)
	Numbers	Percentage (%)	Numbers	Percentage (%)		
*1) Gender*						
Male	46	56.8	35	43.2	Ref.	Ref.
Female	34	61.8	21	38.2	0.6	1.2 (0.61–2.48)

*2) Age*						
< 3 yrs.	39	65.0	21	35.0	Ref.	Ref.
3–4 yrs.	12	41.4	17	58.6	0.04^*∗∗∗*^	0.4 (0.15–0.94)
4–5 yrs.	10	76.9	3	23.1	0.4	1.8 (0.44–7.24)
5 yrs. +	19	55.9	15	44.1	0.3	0.7 (0.29–1.61)

*3) Category of school*						
Public school	54	62.1	33	37.9	Ref.	Ref.
Private school	26	53.1	23	47.0	0.3	0.7 (0.34–1.40)

*4) Grade*						
Playgroup	42	61.8	26	38.2	Ref.	Ref.
Nursery	13	52.0	12	48.0	0.4	0.7 (0.27–1.69)
L.K.G.	12	66.7	6	33.2	0.7	1.2 (0.41–3.70)
U.K.G.	13	52.0	12	48.0	0.4	0.7 (0.27–1.7)

*5) Fathers' education*						
Primary	12	57.1	9	42.9	Ref.	Ref.
Secondary	15	57.7	11	42.3	0.97	1.02 (0.3–3.3)
Higher	20	51.3	19	48.7	0.6	0.8 (0.27–2.30)
Bachelor's degree and above	30	69.8	13	30.2	0.3	1.7 (0.59–5.11)
Uneducated	3	42.9	4	57.1	0.5	0.6 (0.1–3.2)

*6) Mothers' education*						
Primary	12	60.0	8	40.0	Ref.	Ref.
Secondary	14	51.9	13	48.2	0.6	0.7 (0.22–2.31)
Higher	23	56.1	18	43.9	0.8	0.9 (0.29–2.53)
Bachelor's degree and above	23	59.0	12	41.0	0.7	(0.41–3.97)
Uneducated	8	61.5	5	38.5	0.9	1.1 (0.25–4.46)

*7) Class occupancy*						
<15	18	50.0	18	50.0	Ref.	Ref.
15–30	36	70.6	15	29.4	0.05^*∗∗*^	2.4 (0.99–5.84)
>30	26	53.0	23	47.0	0.8	1.1 (0.48–2.67)

*8) Ward*						
Ward no.10	26	54.2	22	45.8	Ref.	Ref.
Ward no.8	18	48.7	19	51.4	0.6	0.8 (0.34–1.89)
Ward no. 7	36	70.6	15	29.4	0.09	2.03 (0.89–4.65)

*9) Antibiotic consumption within 6 months*						
Yes	26	47.3	29	52.7	Ref.	Ref.
No	54	66.7	27	33.3	0.03^*∗∗*^	2.2 (1.1–4.5)

**Table 4:** Multiple logistic regression analysis of bacterial coexistence.

Covariate		*S. aureus*	*S. pneumonia*	*H. influenzae*
*M. catarrhalis*	N (%)	39 (50.0)	39 (70.9)	1 (100)
	Β	−0.747	0.866	16.833
	aOR (95% CI)	0.474 (0.23-0.96)	2.38 (1.15–4919)	2044904892

## Data Availability

The data are available from the corresponding author on reasonable request.

## Abbreviations

AOM: Acute otitis media
C.I: Confidence interval
CAP: Community acquired pneumonia
DNase: Deoxyribonucleases
*H. influenzae*: *Haemophilus influenzae*
IMRAD: Introduction, methods, results, and discussion
L.K.G: Lower kindergarten
LRTI: Lower respiratory tract infection
*M. catarrhalis*: *Moraxella catarrhalis*
NHRC: Nepal Health Research Council
O.R: Odds ratio
PCV: Pneumococcal conjugated vaccine
*S. aureus*: *Staphylococcus aureus*
*S. pneumoniae*: *Streptococcus pneumoniae*
SPSS: Statistical Package for the Social Sciences
U.K.G: Upper kindergarten.
